# On the number of degenerate simplex pivots

**DOI:** 10.1007/s10107-026-02349-x

**Published:** 2026-03-23

**Authors:** Kirill Kukharenko, Laura Sanità

**Affiliations:** 1 Otto von Guericke University Magdeburg, Magdeburg, Germany; 2 Bocconi University, Milan, Italy

**Keywords:** Simplex Algorithm, Linear Programming, Degeneracy

## Abstract

The simplex algorithm is one of the most popular algorithms to solve linear programs (LPs). Starting at an extreme point solution of an LP, it performs a sequence of basis exchanges (called pivots) that allows one to move to a better extreme point along an improving edge-direction of the underlying polyhedron. A key issue in the simplex algorithm’s performance is *degeneracy*, which may lead to a (potentially long) sequence of basis exchanges which do not change the current extreme point solution. In this paper, we prove that one can employ any improving feasible direction at an extreme point to limit the number of consecutive degenerate pivots that the simplex algorithm performs to $n-m-1$, where *n* is the number of variables and *m* is the number of equality constraints of a given LP in standard equality form.

## Introduction

The simplex algorithm first introduced in [14] has always been one of the most widely used algorithms for solving linear programming problems (LPs). Although it performs as a linear time algorithm for many real world linear programming models (see, e.g., the survey [39]) and a variant of it (the shadow simplex) has been shown to have polynomial smoothed complexity (see, e.g., [7, 8, 12, 28, 40, 46]), no polynomial version of the simplex algorithm has been found until these days, despite decades of research.

The simplex algorithm relies on the concept of *basis*, where a basis corresponds to an inclusion-wise minimal set of tight constraints which defines an extreme point solution of the underlying linear program. In each step, it performs a basis exchange by replacing one constraint currently in the basis with a different one that is not in the basis. Such an exchange is called a *pivot*. From a geometric perspective, a basis exchange identifies a direction to move from the current extreme point. The simplex algorithm only considers pivots that yield an *improving* direction, with respect to the objective function to be optimized. As the result of pivoting, it is possible for the algorithm to either stay at the same extreme point, or to move to an adjacent extreme point of the underlying polyhedron. We refer to a pivot of the former type as *degenerate*, in contrast to a pivot of the latter type, which will be called *non-degenerate*. The *pivot rule* which determines how to perform these basis exchanges (i.e., deciding which inequality enters and which inequality leaves the basis) is the core of the simplex algorithm. Various pivot rules have been introduced over decades, but none of them yields a polynomial time version of the simplex algorithm so far (see, e.g., [3, 16, 18, 19, 21, 26, 41, 47] and the references therein).

When analyzing the performance of the simplex algorithm, one can often assume that the underlying polyhedron $\mathcal P$ is non degenerate, e.g., by performing a small perturbation of the constraint’s right-hand-side. In this case, a necessary condition to guarantee an efficient performance of the simplex algorithm, is that there is always a way to reach an optimal solution within a polynomial number of non-degenerate pivots. Geometrically, this would imply that there is always a path between two extreme points on the 1-skeleton of $\mathcal P$ (the graph where vertices correspond to the extreme points of $\mathcal P$, and edges correspond to the 1-dimensional faces of $\mathcal P$), whose length is bounded by a polynomial in the input description of $\mathcal P$. This is a major and still open question in the field, commonly referred to as the polynomial Hirsch Conjecture (see for example [6, 13, 27, 37, 43]).

On the other hand, even in the case when a short path exists, it is not clear how the simplex algorithm would be bound to follow it (see for instance the case of 0/1-polytopes discussed in [4], or the hardness results in [9, 15]). In fact, given any LP one can apply some transformation to it to ensure the existence of a short path between an initial extreme point solution and an optimal one in the underlying polyhedron (such as, e.g., relying on an extended formulation with one extra dimension to construct a pyramid whose apex is adjacent to all extreme points of the initial polyhedron). Such standard transformations usually make the polyhedron become highly degenerate. In the presence of degeneracy, an extreme point might have exponentially many distinct bases defining it, and the simplex algorithm might perform an exponential number of consecutive degenerate pivots, staying in the same vertex, (see, e.g., [11]). Such behavior is referred to as *stalling*. In some pathological cases, certain pivot rules might even provide an infinite sequence of consecutive degenerate pivots at a degenerate vertex, a phenomenon called *cycling*. Although cycling can be easily avoided by employing, for instance, Bland’s rule [5] or a lexicographic rule [42], there is no known pivot rule that prevents stalling. As stated in several papers (e.g., [29, 30]), it is well known that solving a general LP in strongly polynomial time can be reduced to finding a pivot rule that prevents stalling in general polyhedra, and that can be implemented in strongly polynomial time. However, there are a few cases in which it is known that the issue of stalling can be handled. The most famous example is the class of transportation polytopes, for which pivot rules with polynomial bounds on the number of consecutive degenerate pivots were introduced in [11] and further developed in [1, 20, 35]. See also the work of [33] for a strongly polynomial version of the primal network simplex.

Our findings are inspired by [22] to a great extent. The latter work shows that, given an LP in standard equality form with a totally unimodular coefficient matrix $A \in \mathbb \{-1,0,1\}^{m \times n}$, a vertex solution *x* and an improving feasible *circuit direction* for *x*, one can construct a pivot rule which performs at most *m* consecutive degenerate pivots. We generalize their proof, and show that one can employ *any* improving feasible direction at a vertex *x* of a *general* polyhedron, to avoid stalling. Our result, proved in Section 2, is summarized in the following theorem.

### Theorem 1

Given any LP of the form $\min \{c^T x: Ax=b, x\ge 0\}$ with $A \in \mathrm {\hspace{0.1mm}I\hspace{-0.8mm}R}^{m \times n}$, there exists a pivot rule that limits the number of consecutive degenerate simplex pivots at any non-optimal extreme solution to at most $n-m-1$.

The pivot rule can be efficiently implemented whenever an improving feasible direction at a non-optimal extreme point is available.

We stress here that the pivots considered in Theorem 1 are degenerate *simplex* pivots, meaning that each of them yields an *improving* direction at the given extreme point, though this direction is not feasible. It is important to point this out as in general, given two adjacent extreme points $x, x'$, one can easily perform a sequence of basis exchanges that yields $x'$ from *x* by identifying the common tight linearly independent constraints, and introducing each of them to the current basis in any order, until the direction $x'-x$ is seen. However, we here want a strategy that guarantees that *each* basis exchange defines an improving direction, and hence is realizable by the simplex algorithm.

We then discuss some byproducts of our result in Section 3. In particular, we revise the analysis of the simplex algorithm by Kitahara and Mizuno [24, 25] who bound the number of non-degenerate pivots in terms of *n*, *m* and the maximum and the minimum non-zero coordinate of a basic feasible solution. Their analysis combined with our degeneracy-escaping technique show that the *total* number of simplex pivots (both degenerate and non-degenerate) can be bounded in a similar way. As a consequence, one can have a strongly-polynomial number of simplex pivots for several combinatorial LPs. In addition, we perform some computational experiments to evaluate the performance of the antistalling pivot rule in practice, reported in Section 4.

Of course the drawback of the whole machinery is that it requires an improving feasible direction at a given vertex. Though it is efficiently computable, this is in general as hard as solving an LP. For some classes of polytopes though (e.g., matching or flow polytopes) finding such a direction can be easier, thus making it worthwhile to apply our pivot rule. Most importantly, we think that the main importance of our result is from a theoretical perspective: it shows that, for several polytopes, not only a short path on the 1-skeleton exists, but a short sequence of *simplex pivots* always exists (and can also be efficiently computed). That is, a short sequence of *basis exchanges* that follow *improving* directions, when performing *both* degenerate and non-degenerate pivots.

## Antistalling pivot rule

The goal of this section is to prove Theorem 1. Before doing that, we state some preliminaries, and also give a geometric intuition behind the result.

### Preliminaries

The simplex algorithm works with LPs in standard equality form:

1 $$\begin{aligned} \begin{array}{rll} \min &  c^Tx &  \\ \text{ s.t. } &  Ax & = b\\ &  x & \ge 0 \end{array} \end{aligned}$$

For $A \in \mathrm {\hspace{0.1mm}I\hspace{-0.8mm}R}^{m \times n}$ and $B \subseteq [n] {:=\{1,2, \dots , n\}}$, we use $A_B$ to denote the submatrix of *A* formed by the columns indexed by *B*. Similarly, for $x\in \mathrm {\hspace{0.1mm}I\hspace{-0.8mm}R}^n$ and $B\in [n]$, the notation $x_B$ is used to denote a vector consisting of the entries of *x* indexed by *B*. A *basis* of (1) is a subset $B \subseteq [n]$ with $|B| = m$ and $A_B$ being non-singular. The point *x* with $x_B = A_B^{-1}b$, $x_{N} = 0$ where $N:=[n]\setminus B$ is a *basic solution* of (1) with basis *B*. If additionally $x_B \ge 0$, both the basic solution *x* and the basis *B* are *feasible*. If $x_i > 0$ for all $i \in B$, then *B* and *x* are called *non-degenerate*. If instead $x_i=0$ for some $i \in B$, then *B* and *x* are called *degenerate*. We let $\overline{A} := A_B^{-1}A_N$ and $\overline{c}_N^T := c_N^T - c_B^T \overline{A}$. In particular, $\overline{c}_N\in \mathrm {\hspace{0.1mm}I\hspace{-0.8mm}R}^N$ is the vector of so-called *reduced costs* for the basis *B*. The coordinates of the reduced cost vector will be addressed as $\overline{c}_{N,i}$, where the first subscript will be dropped if the basis $B = [n] \setminus N$ is clear from the context.

The simplex algorithm considers in each iteration a feasible *B*. If all elements of $\overline{c}_N$ are non-negative, then the basis *B* and the corresponding basic feasible solution *x* are known to be optimal. Otherwise, the algorithm *pivots* by choosing a non-basic coordinate with negative reduced cost to enter the basis, say *f*. It then performs a minimum ratio test to compute an index *i* that minimizes $\frac{x_i}{\overline{A}_{if}}$ among all indices $i \in B$ for which $\overline{A}_{if} >0$. Such index *i* will be the one leaving the basis, and it corresponds to a basic coordinate which hits its non-negativity bound first when moving along the direction given by the tight constraints indexed by $B\setminus \{f\}$. At each iteration there could be multiple candidate indices for entering the basis (all the ones with negative reduced cost), as well as multiple candidate indices to leave the basis (all the ones for which the minimum ratio test value is attained). The choice of the entering and the leaving coordinates is the essence of a pivot rule (see [41] for a survey). A pivot is *degenerate* if the attained minimum ratio test value is 0 (hence, the extreme point solution *x* does not change), and *non-degenerate* otherwise. Note that only coordinates with negative reduced cost are considered for entering the basis, since only such a choice guarantees the simplex algorithm to make progress when a non-degenerate pivot occurs. To later emphasize that we only refer to that kind of *improving* pivots, we will call them *simplex pivots*.

Finally, we let $\textrm{kern}(A):= \{y \in \mathrm {\hspace{0.1mm}I\hspace{-0.8mm}R}^n: A y =0\}$. Given (1), we call a vector $y \in \textrm{kern}(A)$ with $c^Ty < 0$ an *improving direction*. Such *y* is said to be *feasible* at a basic feasible solution *x* if $x+\varepsilon y \ge 0$ for sufficiently small positive $\varepsilon$. Note that for any $y \in \textrm{kern}(A)$ and any basis *B* of *A*, the following holds:

2 $$\begin{aligned} c^Ty= c^T_By_B + c^T_Ny_N = c^T_B(-\overline{A}y_N) + c^T_Ny_N = \overline{c}_N^Ty_N \end{aligned}$$

### A geometric intuition

**Fig. 1:**
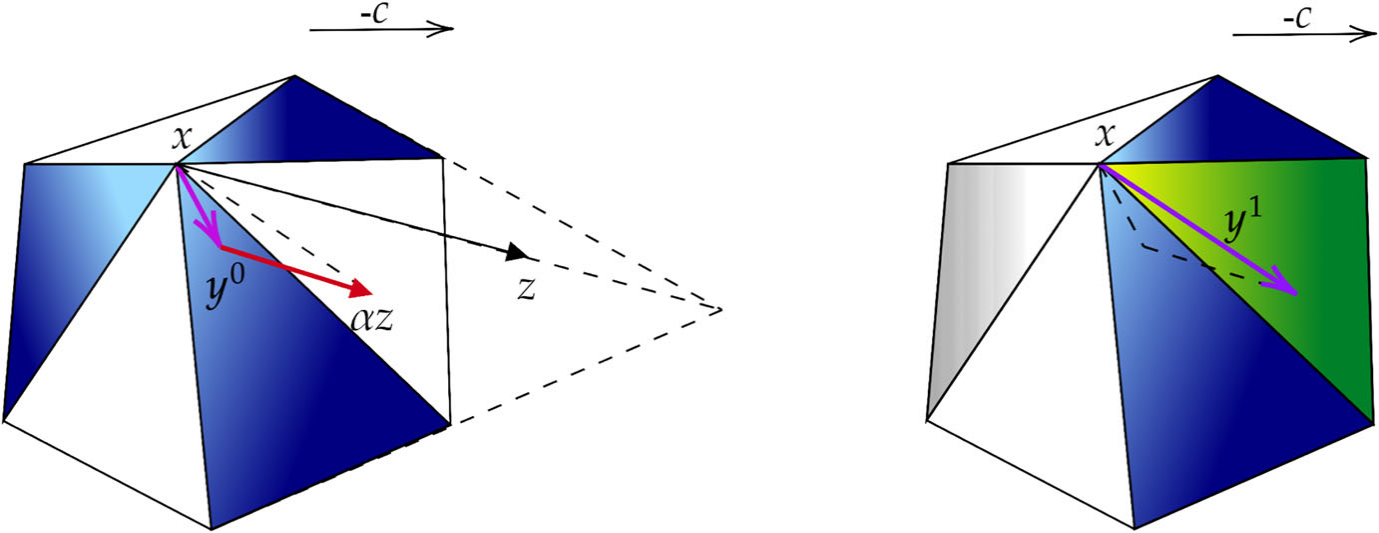
To the left, facets corresponding to inequalities in *N* and *W* are colored in navy and white, respectively. To the right, the gray fading facet is defined by the $f^{th}$ inequality and the green one corresponds to the $g^{th}$

Before providing a formal proof, we give a geometric intuition on how our pivot rule works. For this, it will be easier to abandon the standard equality form. In particular, consider a degenerate vertex $x \in \mathrm {\hspace{0.1mm}I\hspace{-0.8mm}R}^d$ of a polytope $\mathcal P$ of dimension $d \in \mathbb{N}$ defined by inequalities $a_i^Tx \le b_i$ with $a_i \in \mathrm {\hspace{0.1mm}I\hspace{-0.8mm}R}^d, b_i \in \mathrm {\hspace{0.1mm}I\hspace{-0.8mm}R}, i \in [n]$. In this setting, degeneracy means that more than *d* inequalities are tight at *x*. Consider a subset *N* of the set of indices of all inequalities that are tight at *x* with $|N| = d$ and let $B:=[n]\setminus N$ (see Figure 1). Note that here we intentionally redefine a notation used in the standard equality form, where non basic coordinates always have their corresponding constraints tight. Since *x* is degenerate, there is at least one inequality that is tight at *x* with index in *B*. We denote the subset of such inequalities by $W \subseteq B$. Finally, assume *x* is not an optimal vertex of $\mathcal P$ when minimizing an objective function $c \in \mathrm {\hspace{0.1mm}I\hspace{-0.8mm}R}^d$ over $\mathcal P$, and let $y^0 \in \mathrm {\hspace{0.1mm}I\hspace{-0.8mm}R}^d$ be an improving feasible direction at *x*, i.e., such that $x+\varepsilon y^0 \in \mathcal P$ for a sufficiently small positive $\varepsilon$ and $c^Ty^0 < 0$. In order to find an improving edge of $\mathcal P$ at *x*, we look at the directions given by the extremal rays of the basic cone $C(N) := \{x\in \mathrm {\hspace{0.1mm}I\hspace{-0.8mm}R}^n \mid a_i^T x \le 0, i \in N\}$. If there is an improving feasible direction among them, we are done. Otherwise, we reduce the dimension of the polytope in the following way. We pick an extremal ray $z \in C(N)$ such that $c^Tz < 0$. Note that *z* is formed by $d-1$ inequalities from *N*. Let $f \in N$ be the only inequality index not used to define *z*, among the *d* tight inequalities in *C*(*N*) (*f* is depicted as the gray fading facet in Figure 1). Consider a vector combination $y^0 + \alpha z$ where $\alpha \ge 0$ and note that it provides an improving direction for any positive $\alpha$. Since $y^0$ is contained in the feasible cone $C(N\cup W) = \{x\in \mathrm {\hspace{0.1mm}I\hspace{-0.8mm}R}^n \mid a_i^T x \le 0, i \in N \cup W\}$ but *z* is not, the vector $y^0 + \alpha z$ leaves $C(N\cup W)$ for sufficiently large $\alpha$. Hence there has to be a number $\alpha ^1 \ge 0$ such that $y^0 + \alpha ^1 z$ is contained in a facet of $C(N\cup W)$. Without loss of generality assume that the latter facet is defined by the $g^{th}$ inequality (green facet in Figure 1). Note that $g\in W$ since $y^0$ and *z* are both contained in the basic cone *C*(*N*) and so is $y^0 + \alpha z$ for any non-negative $\alpha$. Then, we define a new improving feasible direction $y^1 := y^0 + \alpha ^1 z$, $N = \big (N\setminus \{f\}\big )\cup \{g\}$, $B = \big (B \setminus \{g\}\big ) \cup \{f\}$ and continue searching for an improving edge inside the facet defined by the $g^{th}$ inequality. Since $x+\varepsilon y^1$ belongs to this facet for an $\varepsilon >0$ small enough, the l atter yields dimension reduction. After at most $d-1$ such steps we are bound to encounter a one-dimensional face of $\mathcal P$ containing $x+\varepsilon y^{\prime }$, where $y^{\prime }$ is an improving feasible direction at *x*. For an illustration see Figure 1.

### Proof of Theorem 1

#### Proof

Let *x* be a degenerate basic feasible solution of (1) with basis *B*. Without loss of generality, assume $B = [m]$. Then, $x_i=0$ for $i \in N = [n]\setminus [m]$. Assume also that $x_i = 0$ for $i\in [k]$ with $1 \le k \le m$ and $x_i>0$ for $i=k+1,\dots ,m$. Let $S(B):= \{i \in N \mid \overline{c}_i < 0\}$. Since *x* is not optimal, $S(B) \ne \emptyset$.

It is well known that a basic feasible solution *x* of (1) with basis *B* is not optimal if and only if there exists an improving feasible direction, i.e., there exists $y^0 \in \mathrm {\hspace{0.1mm}I\hspace{-0.8mm}R}^n$ such that $Ay^0= 0$, $c^Ty^0 < 0$ and $y^0_i \ge 0$ for all $i \in [k] \cup \big ([n]\setminus [m]\big )$. Consider any such $y^0$. Let $Q_1(y^0, B):=\{i\in [k]\mid y^0_i > 0\}$ and $Q_2(y^0, B):=\{i\in [n]\setminus [m] \mid y^0_i > 0\}$. Without loss of generality, let $Q_1(y^0, B) = [r]$ with $0 \le r \le k$ and $Q_2(y^0, B) = \{m+1,\dots ,m+t\}$ with $1\le t \le n-m$. See Table 1 for an illustration.

**Table 1:** An illustration of entries of $x, y^0, z$ and $y^1:=y^0+\alpha z$. A positive, a non-negative, a negative, and a sign-arbitrary entry is denoted by +, $0_+$, − and $\star$, respectively. Without loss of generality, we here assumed that the entering index *f* is $m+1$, and that an index *g* for which the minimum in **Case III** is attained is 1.

	1		...	*r*		...	*k*		...	*m*	*f*		...	$m+t$		...	*n*
*x*	0	0	...	0	0	...	0	+	...	+	0	0	...	0	0	...	0
$y^0$	+	+	...	+	0	...	0	$\star$	...	$\star$	+	+	...	+	0	...	0
*z*	–	$\star$	...	$\star$	$0_+$	...	$0_+$	$\star$	...	$\star$	1	0	...	0	0	...	0
$y^0 +\alpha z$	0	$0_+$	...	$0_+$	$0_+$	...	$0_+$	$\star$	...	$\star$	+	+	...	+	0	...	0

Since $c^Ty^0 = \overline{c}_N^T y_N^0 < 0$ due to (2), it follows that $S(B) \cap Q_2(y^0, B) \ne \emptyset$. We choose the entering index to be a non-basic one with the most negative reduced cost^1^ in the support of $y^0$, that is $f = \textrm{argmin}_{i \in S(B) \cap Q_2(y^0, B)} \overline{c}_i$. To detect the leaving index, we consider the following case distinction. Note that the case distinction depends on the basis *B* and the improving feasible direction $y^0$.

**Case I:** *There is no* $i\in [k]$ *with* $\overline{A}_{if} > 0$. In this case, the minimum ratio test for *f* is strictly positive, that is:

$$(*) \quad \min _{i \in Eq^>(f)} \frac{x_i}{\overline{A}_{if}}>0 \text{ where } Eq^>(f) := \{i\in [m] \mid \overline{A}_{if} > 0\}\,.$$

In particular, this means $Eq^>(f) \subseteq [m]\setminus [k]$. We perform a non-degenerate pivot, by selecting an index that minimizes $(*)$ as the leaving index.

**Case II:** $\overline{A}_{if} > 0$ *for some* $i \in [k]\setminus [r]$. In this case, we perform a degenerate pivot by selecting *i* as the leaving index. Let $B^\prime := B \cup \{f\} \setminus \{i\}$. Because of degeneracy, the basic solution associated with $B'$ is still *x*, and hence $y^0$ is still an improving feasible direction for *x*. Note that $|Q_2(y^0, B^\prime )| = |Q_2(y^0, B)| - 1$ since $y^0_f > 0$ but $y^0_i = 0$ by definition. Repeat the same for $B^\prime$ and $y^0$.

**Case III:** $\overline{A}_{if} \le 0$ *for all* $i \in [k]\setminus [r]$ *and* $\overline{A}_{if} > 0$ *for some* $i \in [r]$. In this case, we are going to change our improving feasible direction. Consider the following vector $z \in \mathrm {\hspace{0.1mm}I\hspace{-0.8mm}R}^n$ which is, in fact, an improving (though not feasible) direction for the entering variable $x_f$:

3 $$\begin{aligned} z_i={\left\{ \begin{array}{ll} -\overline{A}_{if} &  \text { for } i\in [m],\\ 1 &  \text { for } i=f,\\ 0 &  \text { otherwise.} \end{array}\right. } \end{aligned}$$

Note that $Az = 0$ and $c^Tz = \overline{c}_f < 0$. Moreover $z_i \ge 0$ for each $i \in \big ([r]\setminus Eq^>(f)\big ) \cup \big ([k]\setminus [r]\big ) \cup \big ([n]\setminus [m]\big )$ and $z_i <0$ for $i \in Eq^>(f)$ by definition. Set

$$y^1:=y^0 + \alpha z \; \text{ with } \; \alpha := \min _{i \in Eq^>(f) \cap [r]} \frac{y_i^0}{-z_i} > 0\,.$$

Observe that $y^1$ is a feasible direction for *x*, since $A(y + \alpha z) = 0$, and $y^1_i \ge 0$ for $i \in [k]\cup \big ([n]\setminus [m]\big )$, because of the choice of *z* and $\alpha$. Furthermore, $y^1$ is an improving direction, because $c^Tz < 0$ and hence

4 $$\begin{aligned} c^Ty^1 = c^Ty^0 + \alpha c^Tz< c^Ty^0 < 0\,. \end{aligned}$$

Note that $Q_2(y^1, B) = Q_2(y^0, B)$. Furthermore, let $g \in Eq^>(f) \cap [r]$ be the index for which the value of $\alpha$ is attained. We have $y^1_g = 0$ and $\overline{A}_{gf}>0$. We now repeat the case distinction above for *B* and $y^1$.

The key observation is that when the third case of the above case distinction has occurred, repeating the same for *B* and $y^1$ falls into the second case (because the basis *B* has not changed, meanwhile $y^1_g = x_g = 0$ with $g \in B$ and $\overline{A}_{gf}>0$). Therefore, after each degenerate pivot, the cardinality of the support of the improving feasible direction in the non-basic indices decreases. Hence, a sequence of $|Q_2(y^0,B)|$ degenerate pivots would yield an improving feasible direction $y'$ and a basis $B'$ of *x* with $Q_2(y', B') = 0$, which in turn implies $0 = \overline{c}^T_{N'}y'_{N'} = c^Ty'$ due to (2), yielding a contradiction. Hence the number of consecutive degenerate pivots with the suggested pivot rule cannot exceed $n-m-1$. $\square$

For the sake of clarity, the antistalling pivot rule is formally summarized in Algorithm 1. 
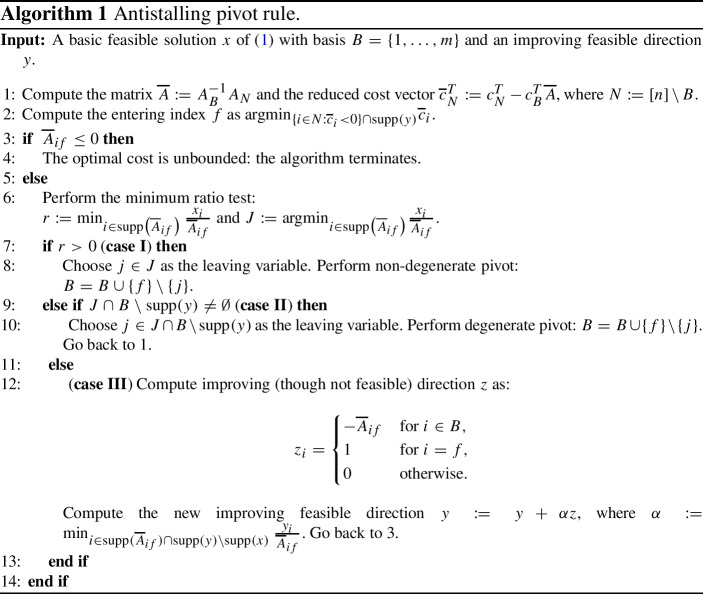


We conclude with a strengthening of the bound of Theorem 1 which will be useful in the next section.

#### Theorem 2

Given any LP of the form $\min \{c^T x: Ax=b, x\ge 0\}$ with $A \in \mathrm {\hspace{0.1mm}I\hspace{-0.8mm}R}^{m \times n}$, there exists a pivot rule that limits the number of consecutive degenerate simplex pivots at any non-optimal extreme solution *x* to at most $\min \{n-m-1, m-1\}$.

The pivot rule can be efficiently implemented whenever an improving feasible direction of the form $\tilde{x}-x$, for some extreme point solution $\tilde{x}$, is available.

#### Proof

Let *x* be the currently considered basic feasible solution with basis *B*. If $y^{0}$ is chosen to be equal to $\tilde{x}- x$ for some basic feasible solution $\tilde{x}$ of our LP with $c^T \tilde{x} < c^T x$, then one can observe that $|Q_2(y^0, B)| \le m$ because $\tilde{x}$ has at most *m* non-zero coordinates. By the proof of Theorem 1 above, the latter value strictly decreases with each degenerate step produced by the antistalling pivot rule. Hence, the total number of consecutive degenerate pivots at the vertex *x* can be strengthened to be at most $\min \{n-m-1, m-1\}$. $\square$

## Exploiting the antistalling rule for general bounds

Here we combine the result of the previous section with the analysis of [24, 25]. The authors of the latter works give a bound on the number of non-degenerate simplex pivots that depends on *n*, *m* and the maximum and the minimum non-zero coordinate of basic solutions. The combined analysis yields a similar bound on the total number of (both degenerate and non-degenerate) simplex pivots.

We need a few additional notations. For any vector *x*, we let $\textrm{supp}(x)$ denote its support. We denote the smallest and the largest non-zero coordinate of any basic feasible solution of (1) by $\delta$ and $\varDelta$, respectively. We let $x^\star$ be an optimal basic feasible solution of (1). Finally, for a generic iteration *q* of the simplex algorithm with basis $B^q$ and an improving feasible direction $y^q$, we let $\varDelta ^q_{\overline{c}} := \max _{\{i \in N^q \mid y^q_i > 0\}} -\overline{c}_{N^q, i}$. Note that $\varDelta ^q_{\overline{c}}$ is the absolute value of the reduced cost of the entering variable according to the antistalling pivot rule defined in the previous section when using $y^q$.

We make use of the following result from [25].

### Lemma 1

(Lemma 4 of [25]) If there exists a constant $\lambda >0$ such that

5 $$\begin{aligned} c^T (x^{q+1} - x^\star ) \le \big ( 1-\frac{1}{\lambda }\big )c^T (x^{q} - x^\star ) \end{aligned}$$

holds for any consecutive distinct basic feasible solutions $x^q\ne x^{q+1}$ generated by the simplex algorithm (with any pivot rule), the total number of distinct basic feasible solutions encountered is at most

$$\begin{aligned} (n-m)\Big \lceil \lambda \ln \frac{m\varDelta }{\delta }\Big \rceil \,. \end{aligned}$$

Given (1), apply the simplex algorithm with the antistalling pivot rule described in the previous section. In particular, at a general iteration *t* of the algorithm, let $B^t,x^t$, and $y^t$ be the basis, the basic solution, and the improving feasible direction considered by the algorithm, respectively. Let $N^t := [n]\setminus B^t$. If $x^{t} \ne x^{t-1}$ (i.e., we encounter $x^t$ for the first time, as a result of a non-degenerate pivot) let $y^t:=x^\star - x^t$. Observe that, once this is specified, the improving feasible direction is determined by the antistalling rule for all remaining degenerate pivots at $x^t$.

### Lemma 2

Let $x^t = x^{t+k}$ be the basic feasible solution associated with bases $B^t$ and $B^{t+k}$, and assume $x^{t+k} \ne x^{t+k+1}$. The following holds:

- $$c^Ty^{t+k} \le c^Ty^{t}$$.
- $$y^{t+k}_{i} = y^t_{i}$$ for all $$i \in N^{t+k} \cap \, \textrm{supp}(y^{t+k})$$.

### Proof

The statement in (a) follows from (4). The statement in (b) follows from the construction of our antistalling rule by induction: if Case III never occurs during the *k* degenerate pivots, then $y^{t+k} = y^t$ and the statement holds trivially. Suppose that the situation of Case III appears for $B^{t+j}, y^{t+j}$. The improving feasible direction changes (as $y^{t+j+1} = y^{t+j} + \alpha z$ ), but among the non-basic coordinates this only affects the value of $y^{t+j+1}_f$ (where *f* is the entering index). Immediately after, Case II occurs and *f* gets pivoted in, while a basic index *i* with $y^{t+j+1}_i=0$ gets pivoted out, i.e., $B^{t+j+1} = \big (B^{t+j}\setminus \{i\}\big )\cup \{f\}$ and $N^{t+j+1} = \big (N^{t+j}\setminus \{f\}\big )\cup \{i\}$. Hence, the statement holds. $\square$

The next result, which establishes (5) with $\lambda := \frac{(n-m)\varDelta }{\delta }$ for the simplex algorithm with the antistalling pivot rule, is inspired by [25][Theorem 3].

### Lemma 3

Let $x^t = x^{t+k}$ be the basic feasible solution associated with bases $B^t$ and $B^{t+k}$. Assume $x^{t-1} \ne x^{t}$ and $x^{t+k} \ne x^{t+k+1}$. We have

$$\begin{aligned} c^Tx^{t+k+1}-c^Tx^\star \le \big ( 1 - \frac{\delta }{(n-m)\varDelta } \big ) (c^Tx^{t+k}-c^Tx^\star )\,. \end{aligned}$$

### Proof

The optimality gap for $x^{t+k}$ can be bounded as follows:

6 $$\begin{aligned} \begin{array}{rcl} c^Tx^{t+k}-c^Tx^\star &  = &  c^Tx^{t}-c^Tx^\star \\ &  = &  -c^Ty^t \\ &  \le & -c^Ty^{t+k} \\ &  = & -\overline{c}^T_{N^{t+k}}y^{t+k}_{N^{t+k}} \\ &  = &  \sum _{i\in N^{t+k}} -\overline{c}^T_{N^{t+k},i} y^{t+k}_{i} \\ &  = &  \sum _{i\in N^{t+k} \mid y^{t+k}_i>0} -\overline{c}^T_{N^{t+k},i} y^{t+k}_{i} \\ &  \le &  (n-m)\varDelta ^{t+k}_{\overline{c}} \varDelta \,, \end{array} \end{aligned}$$

where we used Lemma 2(a) for the first inequality, and (2) for the third equality. The last equality follows from $y^{t+k}_i \ge 0, i \in N^{t+k}$ which is implied by feasibility of $y^{t+k}$ at $x^{t+k}$. The last inequality follows from $\max _{\{i \in N^{t+k} \mid y^{t+k}_i > 0\}} -\overline{c}_{N^{t+k}, i} =\varDelta ^{t+k}_{\overline{c}}$, the fact that $|N^{t+k}|=n-m$, and because $y^{t+k}_{i} = y^t_{i} = x^\star _i - x^t_i \le \varDelta$ holds for any $i \in N^{t+k} \cap \, \textrm{supp}(y^{t+k})$, using Lemma 2(b).

Let $x^{t+k}_{f}$ denote the entering variable at $(t+k)^{th}$ iteration. Note that $x^{t+k+1}_{f} \ne 0$ since $x^{t+k} \ne x^{t+k+1}$. Then,

$$\begin{aligned} \begin{array}{rl} c^Tx^{t+k} - c^Tx^{t+k+1} & = \overline{c}_{N^{t+k}}^T(x^{t+k} - x^{t+k+1})_{N^{t+k}} \\ & = -\overline{c}_{N^{t+k},f} x^{t+k+1}_{f} \\ & \ge \varDelta ^{t+k}_{\overline{c}} \delta \\ & \ge \frac{\delta }{(n-m)\varDelta }c^T(x^{t+k}-x^\star ) \end{array} \end{aligned}$$

hold, where we used (2) for the first equality and (6) for the last inequality. Rearranging the terms yields the lemma statement.$\square$

Now, combining Lemma 1 and Lemma 3 allows to conclude that the simplex algorithm with the antistalling pivot rule described in Section 2 encounters at most $(n-m)\lceil \frac{(n-m)\varDelta }{\delta }\ln \big (m\frac{\varDelta }{\delta }\big )\rceil$ distinct basic feasible solutions. Since Theorem 2 bounds the number of consecutive non-degenerate steps by $\min \{n-m-1,m-1\}$ (again, due to the choice of the improving feasible direction), combining it with the latter result yields a bound on the total number of pivots required to reach an optimal vertex. However, this bound does not take into account the number of (degenerate) pivots that might have to be performed at an optimal vertex to encounter a basis satisfying the optimality criterion. This is due to the fact that the antistalling pivot rule requires an improving feasible direction, which we do not have at an optimal vertex. Hence, we have to handle this case separately.

### Lemma 4

Let *B* and $B^\star$ be a non-optimal and an optimal basis, respectively, both associated with an optimal basic feasible solution $x^\star$ of (1). We can obtain $B^\star$ from *B* by performing at most $n-m$ degenerate simplex pivots.

### Proof

Since *B* is not optimal, there exists $f\in N$ with $\overline{c}_{N, f} = c^Tz<0$ with *z* as in (3). Observe that there exists $i \in B \cap N^\star$ with $\overline{A}_{if}>0$ and $x_i=0$: otherwise, all coordinates of $z_{N^\star }$ would be non-negative and hence $z \in C_{N^\star }:=\{x\in \mathrm {\hspace{0.1mm}I\hspace{-0.8mm}R}^n\mid Ax=0, x_{N^\star } \ge 0\}$. The latter however contradicts the fact that all extreme rays of the above cone $C_{N^\star }$ have non-negative scalar products $\overline{c}_{N^\star }$ with *c* due to optimality of $B^\star$. Hence one could perform a simplex pivot on *B* with entering variable *f* and leaving variable *i*. Let $B^\prime := \big (B \setminus \{i\}\big ) \cup \{f\}$ and $N^\prime := \big (N \setminus \{f\}\big ) \cup \{i\}$. Note that $\overline{c}_{N^\prime ,i} \ge 0$ and $i\in N^\prime \cap N^\star$. Either $B^\prime$ is a optimal basis and we stop, or there exists $j \in N^\prime \setminus \{i\}$ with $\overline{c}_{N^\prime , j}<0$. In the latter case, however, we can enforce the constraint $x_i = 0$ by removing the variable $x_i$ together with the corresponding column of *A* and entry $c_i$ of *c* from (1). By doing so we obtain a new LP with the number of variables smaller by one that has $B^\prime$ and $B^\star$ as a non-optimal and an optimal basis, respectively, since $\overline{c}_{N^\prime \setminus \{i\}, j}<0$ and $\overline{c}_{N^\star \setminus \{i\}} \ge 0$. We can set $B=B^\prime$ and repeat the process. Since at each iteration a variable with index from $N^\star$ gets removed, after at most $n-m$ iterations $B = B^\star$. $\square$

Since the number of degenerate pivots at an optimal vertex is bounded by $n-m$ by the above lemma, we can state the following result.

### Theorem 3

Given an LP (1) and an initial feasible basis, there exists a pivot rule that makes the simplex algorithm reach an optimal basis in at most

$$\min \{n-m,m\}(n-m)\lceil \frac{(n-m)\varDelta }{\delta }\ln \big (m\frac{\varDelta }{\delta }\big )\rceil $$

simplex pivots.

Observe that for integral *A* and *b* (which can be assumed without loss of generality for rational LPs), one can bound $\varDelta \le ||b||_1\varDelta _{A}$ and $\delta \ge \frac{1}{\varDelta _{A}}$ due to Cramer’s rule, where $\varDelta _{A}$ is the largest absolute value of the determinant of a square submatrix of *A*. Then the next statement is a straightforward corollary of the above theorem.

### Corollary 1

For any basic feasible solution of an LP (1) with integral *A* and *b*, there exists a sequence of at most

$$\min \{n-m,m\}(n-m)\lceil (n-m)\varDelta _{A}^2||b||_1\ln \big (m\varDelta _{A}^2||b||_1\big )\rceil $$

simplex pivots leading to an optimal basis.

### Application to combinatorial LPs

We conclude this section by observing that, using the latter corollary, one can prove the existence of short sequences of simplex pivots (that is, of length strongly-polynomial in *n*, *m*) between any two extreme points of several combinatorial LPs, that is, LPs modeling the set of feasible solutions of famous combinatorial optimization problems. We report a few examples below.

*(a) LPs modeling matching/vertex-cover/edge-cover/stable-set problems in bipartite graphs.* For matchings, the LP maximizes a linear function over a set of constraint of the form $\{A'x \le 1, x \ge 0\}$, where the coefficient matrix $A'$ is the node-edge incidence matrix of an undirected bipartite graph. After putting the LP in standard equality form by adding slack variables, we get constraints of the form $\{Ax = 1, x \ge 0\}$, where *A* is a totally unimodular matrix (and so $\varDelta _A =1$). The result then follows from Corollary 1. The same holds for vertex-cover (minimizing a linear function over constraints of the form $\{{A'}^Tx \ge 1, x \ge 0\}$), edge-cover (minimizing a linear function over constraints of the form $\{{A'}x \ge 1, x \ge 0\}$), stable-set (maximizing a linear function over constraints of the form $\{{A'}^Tx \le 1, x \ge 0\}$).

*(b) LPs modeling optimization over the* fractional *matching/vertex-cover/edge-cover/stable-set polytopes.* These correspond to the natural LP relaxations of the problems discussed in (a), for general graphs. The same LPs as in (a), in non-bipartite graphs, have a constraint matrix $A'$ (resp. ${A'}^T$) that is not totally unimodular. However, the set of constraints defines half-integral polytopes (see [2, 31]). Note that, after putting the LPs in standard equality form, the slack variables can be loosely bounded from above by the number of variables *n*. Hence, $\varDelta \le n$ and $\delta \ge \frac{1}{2}$, and the result follows from Theorem 3.

*(c) LP for the stable marriage problem.* The classical stable marriage problem is defined on a bipartite graph where each node has a strict preference order over the set of its neighbours. One looks for a matching that does not contain any blocking pair, that is, a pair of nodes that mutually prefer each other with respect to their matched neighbour. There is an (exact) LP formulation for the problem [36, 45], that has constraints of the form $\{A'x \le 1, B'x \ge 1, x \ge 0\}$. Here $A'$ is again the node-edge incidence matrix of an undirected bipartite graph, while $B'$ is a matrix that stems from imposing an additional constraint for each edge $\{uv\}$, that essentially prevents $\{uv\}$ from being a blocking pair:

$$x_{uv} + \sum _{w: w>_u v} x_{uw} + \sum _{w: w >_v u } x_{vw} \ge 1$$

In the above expression, $w >_u v$ (resp. $w >_v u$) means that *u* prefers *w* over *v* (resp. *v* prefers *w* over *u*). After putting the LP in standard equality form, the slack variables can be bounded by 1. Hence, $\varDelta = \delta = 1$, and the result follows from Theorem 3.

*(d) LPs modeling various flow problems (such as max flows, min cost flows, flow circulations) with unit (or bounded) capacity values.* Flow problems in capacitated graphs are modeled using LPs with constraints of the form $\{{A'}x = b, l \le x \le u\}$ where the constraint matrix $A'$ is here a node-arc incidence matrix of a directed graph, which is totally unimodular (see [38]). The right-hand-side vectors (*b*, *l*, *u*) can be bounded in terms of the total capacity values. Therefore, assuming these are bounded integers, one can rely on Corollary 1 to get the result, similarly to (a).

One can compute the corresponding pivot sequence for the problems mentioned in (a)-(d) by running the simplex with the antistalling pivot rule. However, one needs to first solve the LP and obtain an optimal basic solution in order to use it to guide the antistalling pivot rule.

We remark that the existence of a strongly polynomial sequence of pivots for general min-cost flows (hence for problems in (d)), was known already, as it follows from the fundamental work of Orlin [33], that applies even in the case of large capacity values. In comparison, our bound is weaker as it requires the capacity values to be bounded.

Instead, despite being known to be solvable in strongly polynomial time, to the best of our knowledge the problems in (a)-(c) were not previously known to admit strongly polynomial bounds on the number of simplex pivots for their natural LP formulations.

## Computational experiments

In this section we report some computational experiments that we conducted to evaluate the performance of the antistalling pivot rule when applied to actual linear programs. The code is now available at https://github.com/kirill-kukharenko/antistalling.

For the implementation, we used the python package CyLP [44] which wraps COIN-OR’s CLP solver [10] and provides tools for implementing a preferred pivot rule in python to substitute CLP’s built-in ones. We used two different testsets of diverse dimensions and sparsity. The first one is the benchmark Netlib LP dataset [32], containing 108 LPs. The second one contains the root LPs of the benchmark set of MIPLIB 2017 [17], and is composed of 240 instances. All our experiments were conducted on a laptop with Intel Core i7-13700H 2.40GHz CPU and 32GB RAM.

For each of the aforementioned LPs, we ran the simplex algorithm with our antistalling pivot rule, and in addition with other 6 well-known pivot rules (which [44] provides implementations for). These are:

- Dantzig’s rule: the entering variable is the one with the most negative reduced cost;
- Steepest edge: the entering variable is the one which yields a direction *z* maximizing $\frac{-c^Tz}{||z||_2}$ (i.e., maximizing the improvement normalized according to the 2-norm);
- LIFO: the entering variable is the one that minimizes the number of iterations that have past since the variable left the basis;
- Most Frequent: the entering variable is the one that maximizes the number of times it was previously chosen as the entering variable;
- Bland’s rule: the entering variable is the one with the lowest index.
- Positive edge rule: the entering variable is selected with a heuristic that tries to identify non-degenerate pivots with high probability [34].

We tracked the number of pivots required by each of the above rules to solve the LPs to optimality (we did not perturb the instances). For our antistalling pivot rule, we guided it using the feasible direction $y=x^\star - x$ at any non-optimal vertex *x* of the LP, for a pre-computed optimal basic solution $x^*$ (in particular, the one found by Dantzig’s pivot rule). Intuitively, this represents the best direction that can possibly guide our rule, as it is the direction leading immediately to an optimal solution. Our objective with the computational experiments is to see whether this choice translates into few pivots in practice, since *y* might change during the degenerate steps and therefore in reality we do not have control on the actual edge-direction we end up moving along.

We remark that we are here interested in the *number of pivots* required by various pivot rules to solve LPs to optimality, and not in the *computational time* needed to do so. In fact, the CyLP package connects to the solver through a relatively slow Python interface, in contrast to implementing the rules directly into a solver. Therefore, the efficiency of the pivot rule implementations, and hence the number of problems solved by each rule within the time/memory limit, can be heavily affected by this overhead. For a fair comparison, in this section we then only included the instances solved to optimality by all respective pivot rules. However, for completeness, detailed tables with all the results of the computational experiments, for both Netlib and MIPLIB testsets, can be found in Appendices A and B, respectively.

**Fig. 2:**
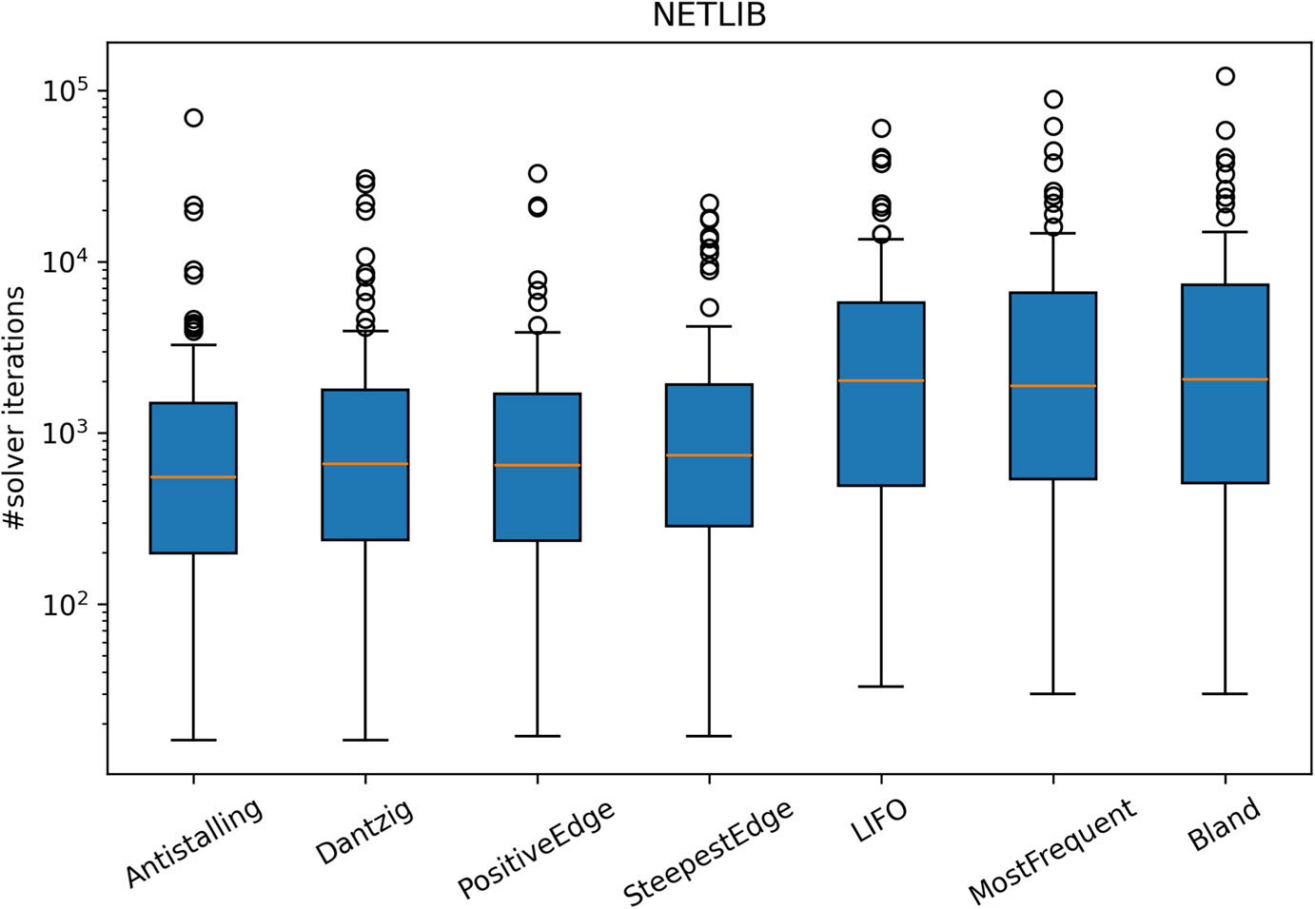
Netlib dataset results. Data are presented using standard *box plots,* which display the distribution of a dataset based on its quartiles. Specifically, the box extends from the first quartile (Q1) to the third quartile (Q3), representing the middle $50\%$ of the data. The thin vertical lines (whiskers) extend from the box to the most extreme data points lying within 1.5 times the interquartile range (Q3-Q1). Points beyond the whiskers are shown individually as outliers (fliers). Orange horizontal lines denote the medians: 551, 659, 648, 739, 2027, 1890, and 2049, respectively

**Fig. 3:**
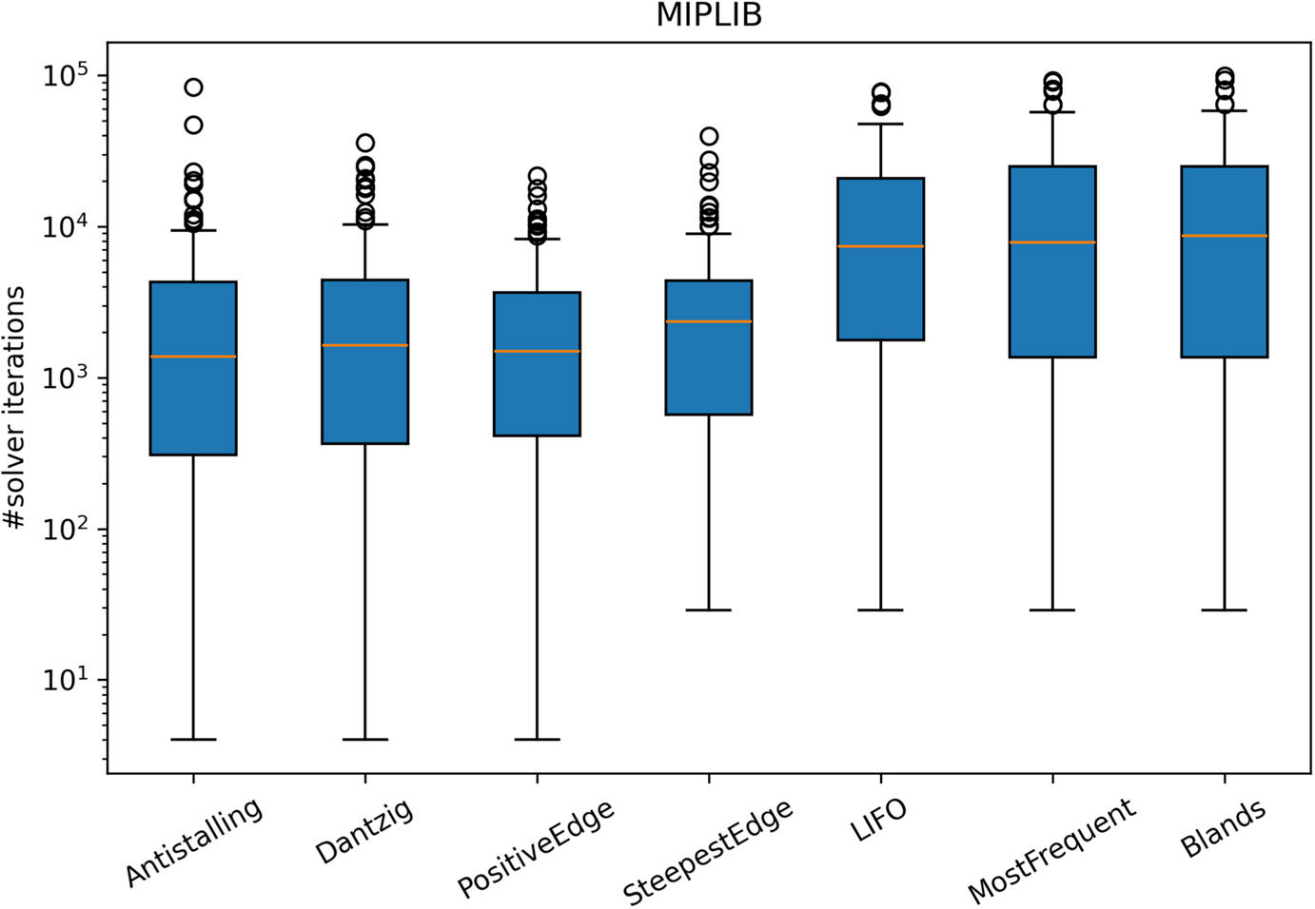
MIPLIB dataset results. Orange lines denote the medians: 1376, 1633, 1493, 2335, 7374, 7829, and 8641, respectively

Our first report deals with 84 out of 108 Netlib problems, for which each of the aforementioned 7 pivot rules was able to find an optimal solution within the time limit of 30 minutes and the memory limit of 32GB RAM. For the results, see Figure 2. Similarly, for MIPLIB this restriction leaves us with 117 out of 240 problems. See Figure 3 for the results.

The computational experiments show that the antistalling pivot rule, guided by a known optimal solution, actually performs quite well, especially on the Netlib instances. Most often it manages to find a relatively short sequence of pivots to an optimal basis compared to the other pivot rules. We highlight for example the problems 25fv47 from Netlib and istanbul-no-cutoff.mps from MIPLIB, where the antistalling pivot rule required less than one fourth of the number of iterations of any other rule. On the other hand, there are instances, e.g., fit2p from Netlib and ic97_potential.mps from MIPLIB, where the antistalling pivot rule actually showed the worst performance compared to all its competitors.

Overall, the results seem to confirm that guidance by an optimal solution indeed often translates into a small number of pivots, motivating further theoretical studies on how such a direction could be approximately followed.

## Final Remarks

We would like to conclude the paper with some remarks and potential research directions that originate from our findings.

The results of this work show the existence of a short sequence of degenerate simplex pivots that leads out of degeneracy. It would be interesting to show that similar results can be achieved through perturbations. Informally, perturbing the right-hand-side of the constraints has the effect of “splitting” a degenerate vertex into a set of (possibly exponentially many) distinct vertices, each associated to a distinct basis. It is legitimate to ask whether every new vertex allows for a short monotone path (where monotonicity is with respect to the linear objective function of the LP), which leads to a vertex out of this set. Note that the existence of the short degeneracy-escaping sequence of simplex pivots showed in this work, generally does not answer the latter question since some bases used in the sequence might become infeasible after perturbation.

Next, we would like to connect the above question to the recent work [23]. Given a basis *B* defining a degenerate vertex *v*, one could imagine to separate *v* from all its neighbouring vertices by a hyperplane: this way, one would obtain a pyramid (with *v* at its apex). Suppose this pyramid is full-dimensional and is described by some $Ax \le b$: then, the above question is equivalent to perturbing the side facets of the pyramid to allow for a short path from the new apex defined by the given basis *B*, to any of the vertices in the bottom of the pyramid. This can be done relying on the techniques used for the construction of a so-called rock extension [23] (when viewing the base of the pyramid as the $d-1$-dimensional original polytope, and the pyramid as its *d*-dimensional extension). Such rock extension has been proven to have the property that the apex is connected to any bottom vertex by a path of at most linear length. However these paths are not necessarily monotone for a given linear objective function, and moreover, the construction of the rock extension requires not only perturbing the right-hand-side *b* but the left-hand-side *A* as well.

Another future research direction, that seems to be supported also by the performed computational experiments, would be to analyze the relation between the improving feasible direction *y* that is required by our antistalling pivot rule, and the actual edge-direction *z* along which one ends up moving after all the degenerate pivots. It would be very interesting to identify some conditions which ensure that *z* is a good approximation of *y*, e.g., in terms of objective function’s improvement.

Furthermore, as it was mentioned in Section 2, in order for our bound to hold, the only requirement we need to impose when performing a pivot is that the entering variable lies in the support of *y* (besides of course having negative reduced cost). In order to obtain the results from Section 3, where both degenerate and non-degenerate pivots have to be taken into account, we chose the entering variable to be the one with the most negative reduced cost, i.e., we used Dantzig’s rule just restricted to the coordinates in $\textrm{supp}(y)$. Of course, one could think of analyzing the performance of the antistalling pivot rule with other choices of entering coordinates from $\textrm{supp}(y)$, e.g., according to steepest edge or shadow vertex. Variations of these latter two rules, in particular, have been shown to play a key role in the context of 0/1 polytopes [4].

## Detailed Netlib results

See Table 2.

**Table 2:** Netlib LPs. * signifies the instance couldn’t be solved by the respective pivot rule within the time and memory limit

problem	Dantzig	PositiveEdge	LIFO	MostFrequent	SteepestEdge	Bland	Antistalling
25fv47.mps	8617	7892	9934	10016	9530	9870	1468
80bau3b.mps	19858	20774	37525	38130	17628	37965	4119
adlittle.mps	111	125	197	316	98	326	56
afiro.mps	16	17	33	30	17	30	16
agg.mps	130	134	300	228	228	233	128
agg2.mps	216	188	210	194	258	200	228
agg3.mps	239	166	317	236	276	249	136
bandm.mps	674	770	2522	2762	1283	2694	674
beaconfd.mps	104	102	117	117	195	117	89
blend.mps	92	86	223	299	122	253	82
bnl1.mps	3290	2228	6295	6408	1883	6412	684
bnl2.mps	12115	*	19314	*	*	*	1524
boeing1.mps	675	695	2485	2570	842	2726	401
boeing2.mps	174	163	518	550	300	568	230
bore3d.mps	198	199	449	496	291	424	152
brandy.mps	353	327	1595	1701	1060	1620	182
capri.mps	506	479	561	1148	416	983	334
cre-a.mps	5828	3202	19458	25810	14105	26623	1554
cre-b.mps	326739	31408	46413	295815	*	322064	10028
cre-c.mps	6712	3504	20850	24339	17947	23915	1483
cre-d.mps	316546	22426	73494	265573	*	281915	38690
cycle.mps	5383	5152	*	*	*	*	4621
czprob.mps	1905	2557	14585	14724	1373	14899	886
d2q06c.mps	29315	29346	30883	*	*	31571	5661
d6cube.mps	22028	21286	21790	22154	22061	21891	21569
degen2.mps	1891	1342	3480	3431	729	3564	627
degen3.mps	10713	5816	12145	12312	2839	11822	4241
dfl001.mps	104120	99785	104924	105564	*	97847	73090
e226.mps	718	561	1790	1820	767	1806	216
etamacro.mps	731	609	2652	*	*	*	995
fffff800.mps	868	671	4169	4922	900	4943	1216
finnis.mps	611	488	810	1385	523	1163	347
fit1d.mps	2636	2369	3301	3290	2532	3377	3254
fit1p.mps	2193	1888	5656	7214	1340	7246	4612
fit2d.mps	29994	31236	31841	31923	*	31880	9417
fit2p.mps	30716	33042	60330	61925	11275	58919	69711
forplan.mps	243	199	*	2065	*	2055	136
ganges.mps	1628	1834	2237	2521	1801	2217	1728
gfrd-pnc.mps	1238	1030	1501	1626	1062	1813	801
greenbea.mps	28646	20538	*	*	*	33255	26251
greenbeb.mps	30340	13041	33436	35418	*	*	30644
grow15.mps	725	647	2964	2217	2455	3016	1524
grow22.mps	1038	904	4612	3410	5427	5263	2909
grow7.mps	306	260	1157	702	838	941	469
israel.mps	331	285	453	747	431	887	123
kb2.mps	61	65	167	127	70	128	55
ken-07.mps	4622	3825	11384	18902	3748	18336	3929
ken-11.mps	31812	29710	113471	114524	*	114656	40987
ken-13.mps	222253	91988	228878	230157	*	229959	*
ken-18.mps	*	*	*	*	*	*	*
lotfi.mps	207	287	1573	1558	295	1653	121
maros.mps	3922	2559	8837	9335	3880	8738	4375
maros-r7.mps	4144	4280	40649	44360	4176	40737	4144
modszk1.mps	1125	1082	4478	7128	1327	7132	672
nesm.mps	4392	4392	*	11946	*	*	1508
osa-07.mps	600	1293	2402	2205	750	4504	1090
osa-14.mps	1400	2510	5553	4787	1500	8807	806
osa-30.mps	2617	4925	11199	14545	*	30277	1551
osa-60.mps	5234	8905	22634	149478	*	78809	2966
pds-02.mps	1776	1668	10942	16084	2099	32573	19604
pds-06.mps	28565	6813	39989	88880	8904	122043	8402
pds-10.mps	122401	13140	65131	154595	*	208463	18440
perold.mps	6339	5416	7558	*	*	7697	1792
pilot.ja.mps	5565	*	10530	*	*	*	1354
pilot.mps	*	*	*	*	*	*	*
pilot.we.mps	8145	3862	14524	14493	13676	14690	9037
pilot4.mps	1326	1636	4895	*	*	*	1409
pilot87.mps	*	*	*	*	*	*	*
pilotnov.mps	1812	2582	12539	12187	12088	12530	907
recipe.mps	48	48	64	64	48	64	48
sc105.mps	103	102	148	120	102	119	108
sc205.mps	231	232	294	252	234	228	224
sc50a.mps	49	45	56	53	49	53	47
sc50b.mps	49	50	51	50	49	50	50
scagr25.mps	653	834	3311	3042	695	3081	771
scagr7.mps	140	186	501	361	190	401	154
scfxm1.mps	457	530	1826	1960	613	1694	537
scfxm2.mps	993	1053	4854	4990	1380	5010	2380
scfxm3.mps	1534	1575	7462	7499	2015	7519	1928
scorpion.mps	364	366	782	444	363	450	382
scrs8.mps	667	809	5386	5334	555	5474	319
scsd1.mps	272	237	2228	2753	180	2697	202
scsd6.mps	620	517	4913	4845	602	4964	237
scsd8.mps	1918	1307	10620	10652	1719	10563	2037
sctap1.mps	317	356	718	822	368	1156	191
sctap2.mps	1225	1270	1095	1352	1533	2077	658
sctap3.mps	1907	1756	1482	1626	2231	1586	944
seba.mps	560	649	508	510	876	507	976
share1b.mps	247	290	1156	1148	211	947	302
share2b.mps	157	131	245	368	156	408	216
shell.mps	733	744	1216	1268	680	1406	779
ship04l.mps	323	343	1649	1637	368	2022	366
ship04s.mps	313	328	1028	1018	346	1159	363
ship08l.mps	598	628	2469	2534	588	3413	540
ship08s.mps	566	552	1328	1335	551	1628	496
ship12l.mps	1111	1017	6077	6234	988	8259	1152
ship12s.mps	951	914	3005	3558	919	2959	881
sierra.mps	1109	1134	970	1029	1864	1020	563
stair.mps	489	507	2884	800	503	728	1842
standata.mps	69	67	118	123	70	127	63
standgub.mps	69	67	118	123	70	127	80
standmps.mps	333	379	470	829	342	999	208
stocfor1.mps	79	79	241	268	79	247	79
stocfor2.mps	2001	2212	13526	13608	3591	13492	1774
tuff.mps	1655	720	2965	2991	701	2945	1011
vtp.base.mps	230	140	381	572	175	513	160
wood1p.mps	665	404	9584	9337	3282	9208	381
woodw.mps	2401	1877	*	32532	*	31839	2491

## Detailed MIPLIB results

See Table 3.

**Table 3:** MIPLIB root LPs. * signifies that the instance couldn’t be solved by the respective pivot rule within the time and memory limit

problem	Dantzig	PositiveEdge	LIFO	MostFrequent	SteepestEdge	Blands	Antistalling
30n20b8.mps	354	413	29832	51208	8610	58208	294
50v-10.mps	282	272	1114	1037	2729	1027	332
CMS750_4.mps	16295	13901	96361	100745	*	101308	41850
academictimetablesmall.mps	40401	26544	171784	176255	*	*	8027
air05.mps	18529	17937	24638	25176	2531	25058	19200
app1-1.mps	2602	2488	11247	22824	7809	22766	3886
app1-2.mps	26462	25375	*	*	*	*	*
assign1-5-8.mps	314	334	1235	1199	496	1149	239
atlanta-ip.mps	*	144325	*	*	*	*	*
b1c1s1.mps	8515	7124	24663	27554	13659	27456	2649
bab2.mps	*	*	*	*	*	*	*
bab6.mps	88009	57985	*	*	*	*	*
beasleyC3.mps	1313	1091	5691	3444	2403	2813	1430
binkar10_1.mps	1519	1493	6279	9385	1714	10168	1376
blp-ar98.mps	750	806	10794	14173	2897	52747	244
blp-ic98.mps	383	353	10432	15684	2125	43764	90
bnatt400.mps	2098	1085	5055	24275	1128	18792	2144
bnatt500.mps	3257	1616	6261	34694	1326	33572	6050
bppc4-08.mps	267	307	5373	4064	3543	3194	203
brazil3.mps	224690	143249	228236	*	*	*	*
buildingenergy.mps	*	*	*	*	*	*	*
cbs-cta.mps	3915	4629	44014	19417	*	19132	3018
chromaticindex1024-7.mps	112861	*	*	*	*	*	*
chromaticindex512-7.mps	53453	*	*	*	*	*	24224
cmflsp50-24-8-8.mps	62624	29725	66646	66879	*	66483	46395
co-100.mps	1810	1432	27194	155300	*	156246	79949
cod105.mps	5043	10413	10806	*	2350	*	3517
comp07-2idx.mps	36630	19996	120086	140436	*	140131	124969
comp21-2idx.mps	17908	11221	77106	90612	3935	99479	83129
cost266-UUE.mps	1612	1782	3310	3582	4375	3626	1827
cryptanalysiskb128n5obj14.mps	83012	94390	*	125246	*	*	*
cryptanalysiskb128n5obj16.mps	83670	91007	*	142608	*	*	*
csched007.mps	1440	1902	7905	8245	1777	7794	603
csched008.mps	1012	1024	6211	6404	1265	6246	1647
cvs16r128-89.mps	28014	28833	26663	25872	*	28176	27912
dano3_3.mps	50506	*	*	*	*	*	*
dano3_5.mps	50506	*	*	*	*	*	*
decomp2.mps	2644	2574	7315	7555	3674	12070	5481
drayage-100-23.mps	4423	2050	15077	27251	2049	38281	6012
drayage-25-23.mps	4599	2189	14278	30007	1812	24166	6277
dws008-01.mps	313	246	1261	527	430	478	358
eil33-2.mps	233	241	1600	2129	151	2469	603
eilA101-2.mps	3325	3514	36453	79566	856	*	56899
enlight_hard.mps	29	29	29	29	29	29	29
ex10.mps	*	179784	*	*	*	*	*
ex9.mps	*	84528	*	*	*	*	*
exp-1-500-5-5.mps	602	1031	639	639	1311	639	457
fast0507.mps	27383	24272	196331	197020	*	197160	22921
fastxgemm-n2r6s0t2.mps	5691	3666	17063	22611	2964	19282	5782
fhnw-binpack4-4.mps	665	667	960	625	1230	615	994
fhnw-binpack4-48.mps	9521	7946	8830	4430	19723	4406	7236
fiball.mps	6688	8910	12339	79670	*	73548	4025
gen-ip002.mps	71	71	147	90	86	91	32
gen-ip054.mps	61	61	152	104	49	105	30
germanrr.mps	3879	4119	*	56995	*	*	9180
gfd-schedulen180f7d50m30k18.mps	*	*	*	*	*	*	*
glass-sc.mps	1589	2876	20767	21471	499	22452	610
glass4.mps	93	86	304	235	116	187	73
gmu-35-40.mps	1651	765	2876	1947	2183	2582	1494
gmu-35-50.mps	1955	1041	3785	3745	2992	4648	2405
graph20-20-1rand.mps	3843	2835	24081	*	2517	12187	4597
graphdraw-domain.mps	268	309	622	304	386	292	168
h80x6320d.mps	364	263	10938	8128	6955	8835	499
highschool1-aigio.mps	*	*	*	*	*	*	*
hypothyroid-k1.mps	10923	11179	26362	15838	7999	24741	4307
ic97_potential.mps	634	598	752	514	636	519	1101
icir97_tension.mps	3186	2145	3432	4216	3587	3547	3383
irish-electricity.mps	*	*	*	*	*	*	*
irp.mps	225	287	*	4075	139	23929	191
istanbul-no-cutoff.mps	11975	11678	82444	86299	*	80471	2781
k1mushroom.mps	81426	16692	18513	5144	*	5400	*
lectsched-5-obj.mps	154064	*	19026	65039	*	64952	45012
leo1.mps	1906	1042	10858	22634	10125	22835	275
leo2.mps	4766	2269	17501	36383	12522	36488	193
lotsize.mps	1918	1785	3498	3567	3148	3599	2010
mad.mps	37	87	97	154	52	226	40
map10.mps	91211	*	*	*	*	*	*
map16715-04.mps	*	*	*	*	*	*	*
markshare2.mps	39	39	97	110	62	107	39
markshare_4_0.mps	20	20	39	42	31	37	19
mas74.mps	129	129	516	566	307	563	23
mas76.mps	79	79	414	500	110	528	37
mc11.mps	1677	1894	3430	2643	4578	2592	1327
mcsched.mps	4708	5116	11932	11842	10099	11784	5820
mik-250-20-75-4.mps	75	75	75	75	75	75	75
milo-v12-6-r2-40-1.mps	3739	5212	26261	15404	4622	9373	2451
momentum1.mps	12355	8999	*	*	*	*	11537
mushroom-best.mps	*	57526	10307	46970	1123	*	*
mzzv11.mps	39840	13789	73458	88323	*	106101	3987
mzzv42z.mps	12448	9094	62218	56988	11379	80630	3614
n2seq36q.mps	4060	2892	77923	81065	3048	79541	4388
n3div36.mps	143	150	2276	5299	295	8577	37
n5-3.mps	1396	995	11634	11481	2012	11550	890
neos-631710.mps	31810	17901	*	*	*	*	*
neos-662469.mps	30106	23253	61914	63061	*	62946	50345
neos-787933.mps	2163	2246	*	9279	*	8700	2164
neos-827175.mps	171542	*	*	165678	*	165157	*
neos-848589.mps	2135	*	*	13758	*	*	2114
neos-860300.mps	1155	893	7698	7829	1321	7938	224
neos-873061.mps	110262	*	*	*	*	*	33
neos-911970.mps	129	133	3183	3363	214	3447	92
neos-933966.mps	158717	167025	169776	155596	*	158554	109968
neos-950242.mps	6928	7299	64874	80748	2590	5921	2630
neos-957323.mps	28302	12481	*	*	*	*	*
neos-960392.mps	9058	10795	203203	205651	*	206419	8805
neos17.mps	936	767	3430	1605	570	1379	700
neos5.mps	105	107	193	122	99	270	7
neos8.mps	712	429	4581	6209	553	9684	1124
neos859080.mps	4	4	93	91	83	90	4
neos-1122047.mps	8685	8825	*	*	*	*	9201
neos-1171448.mps	79780	71487	59813	6001	*	5843	18055
neos-1171737.mps	25462	21642	21740	2542	22777	2630	22927
neos-1354092.mps	90484	88911	67471	67230	*	61909	15903
neos-1445765.mps	6122	6555	28656	31982	10084	46889	5772
neos-1456979.mps	35773	8662	30198	35218	2142	35123	3378
neos-1582420.mps	3670	2964	47584	63690	6191	64109	4293
neos-2075418-temuka.mps	*	*	*	*	*	*	*
neos-2657525-crna.mps	360	200	627	699	305	678	292
neos-2746589-doon.mps	120878	36276	*	271882	*	*	14163
neos-2978193-inde.mps	928	2709	37226	64529	2335	64472	833
neos-2987310-joes.mps	14222	13281	23023	25572	*	26282	15458
neos-3004026-krka.mps	6660	6660	6305	6305	*	6305	11610
neos-3024952-loue.mps	3645	3863	13076	4545	8948	5767	3073
neos-3046615-murg.mps	169	169	235	235	455	235	169
neos-3083819-nubu.mps	2678	2409	8224	11544	2240	15352	1681
neos-3216931-puriri.mps	*	34396	45197	45797	9782	50236	*
neos-3381206-awhea.mps	1584	1774	2196	2312	1978	2227	2002
neos-3402294-bobin.mps	1066	1023	3374	*	461	*	1134
neos-3402454-bohle.mps	5481	3457	*	*	*	*	*
neos-3555904-turama.mps	6076	1607	*	*	*	*	4329
neos-3627168-kasai.mps	1633	1900	4127	3905	5382	5776	1217
neos-3656078-kumeu.mps	20525	17539	113007	110161	*	109498	9260
neos-3754480-nidda.mps	291	430	2202	974	635	1035	231
neos-3988577-wolgan.mps	40469	24658	*	*	*	*	*
neos-4300652-rahue.mps	85331	17034	48186	*	*	*	26589
neos-4338804-snowy.mps	683	692	441	441	792	441	1681
neos-4387871-tavua.mps	2292	2471	9202	6459	3000	26601	3427
neos-4413714-turia.mps	27839	10821	*	*	*	*	16425
neos-4532248-waihi.mps	3191	982	*	*	*	*	7981
neos-4647030-tutaki.mps	11605	12066	18514	18452	*	18167	*
neos-4722843-widden.mps	14983	10825	*	30597	*	37043	*
neos-4738912-atrato.mps	4027	5190	26204	26796	27602	26215	5533
neos-4763324-toguru.mps	302123	71563	*	*	*	*	*
neos-4954672-berkel.mps	962	965	3574	3792	3055	4385	670
neos-5049753-cuanza.mps	*	*	*	*	*	*	*
neos-5052403-cygnet.mps	*	*	*	*	*	*	*
neos-5093327-huahum.mps	19617	20575	*	*	*	*	*
neos-5104907-jarama.mps	*	*	*	*	*	*	*
neos-5107597-kakapo.mps	6191	6969	14446	11175	*	10175	8147
neos-5114902-kasavu.mps	*	*	*	*	*	*	*
neos-5188808-nattai.mps	26125	12571	24862	46541	*	*	9429
neos-5195221-niemur.mps	43996	14654	21534	*	*	*	13985
net12.mps	3929	5043	29848	92171	4716	92870	1160
netdiversion.mps	*	*	*	*	*	*	*
nexp-150-20-8-5.mps	661	650	2707	5219	11536	6470	845
ns1116954.mps	5600	12858	441155	24617	*	81870	19024
ns1208400.mps	3263	2398	25447	25859	2281	26724	4545
ns1644855.mps	*	*	*	*	*	*	*
ns1760995.mps	*	31060	*	*	*	*	*
ns1830653.mps	18120	16096	18666	19139	4744	19311	11198
ns1952667.mps	68	68	11145	18491	87	24957	68
nu25-pr12.mps	1850	1642	6699	26414	3099	26281	6165
nursesched-medium-hint03.mps	156192	36390	160684	169581	*	168361	35869
nursesched-sprint02.mps	16279	8259	47487	46400	3100	46185	46907
nw04.mps	438	313	12328	56821	130	*	94
opm2-z10-s4.mps	*	*	28984	*	*	*	*
p200x1188c.mps	578	579	1603	1061	1177	1018	1244
peg-solitaire-a3.mps	11502	10055	31045	30794	6902	30915	19201
pg.mps	1566	1567	3628	3730	8714	4477	1552
pg5_34.mps	1684	1999	8040	8664	3631	8641	3022
physiciansched3-3.mps	*	*	*	*	*	*	*
physiciansched6-2.mps	*	*	*	*	*	*	*
piperout-08.mps	10035	8071	77316	78677	13883	79658	10936
piperout-27.mps	13212	10491	93333	97329	*	98047	19730
pk1.mps	56	56	234	256	229	220	56
proteindesign121hz512p9.mps	963	905	13935	19537	*	*	1311
proteindesign122trx11p8.mps	606	652	11270	10568	2918	10460	713
qap10.mps	43368	*	47037	42295	*	51073	8507
radiationm18-12-05.mps	40126	50952	*	*	*	*	*
radiationm40-10-02.mps	163087	*	*	*	*	*	*
rail01.mps	*	*	*	*	*	*	*
rail02.mps	*	*	*	*	*	*	*
rail507.mps	24907	20067	195912	196516	*	197922	23777
ran14x18-disj-8.mps	1840	1657	3587	3867	3889	3821	2611
rd-rplusc-21.mps	475	412	342	913	640	840	517
reblock115.mps	2456	5185	11934	2566	4122	2627	3220
rmatr100-p10.mps	3409	4706	35091	15813	1672	17698	501
rmatr200-p5.mps	65929	85034	*	*	*	*	309
rocI-4-11.mps	9498	13144	46230	54614	7263	42979	20292
rocII-5-11.mps	6064	1315	3874	2342	1884	2382	1615
rococoB10-011000.mps	20776	1353	21938	21425	3977	21581	11982
rococoC10-001000.mps	3941	1511	7374	13936	5032	14324	522
roi2alpha3n4.mps	24697	10346	6264	32500	39804	10864	9391
roi5alpha10n8.mps	12459	6064	13999	*	*	132274	*
roll3000.mps	1815	2155	12717	13269	1892	13997	3166
s100.mps	*	*	*	*	*	*	*
s250r10.mps	*	*	*	*	*	*	*
satellites2-40.mps	73896	51346	19366	213784	*	21851	2498
satellites2-60-fs.mps	47644	41964	18033	181057	*	16223	2921
savsched1.mps	*	*	*	*	*	*	*
sct2.mps	6949	2983	27287	26895	7397	27005	3571
seymour.mps	7345	9261	22067	24986	2502	24940	15189
seymour1.mps	7345	9261	22067	24986	2502	24940	15189
sing44.mps	261483	*	*	*	*	*	*
sing326.mps	220981	*	*	*	*	*	*
snp-02-004-104.mps	*	*	*	*	*	*	*
sorrell3.mps	1397	1356	1772	1369	1335	1368	1736
sp150x300d.mps	318	310	577	479	484	475	307
sp97ar.mps	3950	3573	40951	50077	2571	49995	14935
sp98ar.mps	20342	10799	39116	52886	2519	52395	1967
splice1k1.mps	35351	17482	34404	48443	*	32872	16224
square41.mps	*	*	*	*	*	*	*
square47.mps	*	*	*	*	*	*	*
supportcase10.mps	*	*	*	*	*	*	*
supportcase12.mps	*	*	*	*	*	*	*
supportcase18.mps	1787	1077	22628	27455	652	37010	499
supportcase19.mps	*	*	*	*	*	*	*
supportcase22.mps	*	2431	*	*	*	*	*
supportcase26.mps	294	313	365	409	450	409	540
supportcase33.mps	4216	3176	44224	140227	*	87128	5053
supportcase40.mps	100160	38705	*	175363	*	181878	21721
supportcase42.mps	*	*	*	*	*	*	*
supportcase6.mps	9576	3895	*	*	*	*	931
supportcase7.mps	154110	24378	*	*	*	*	*
swath1.mps	640	1049	19946	23793	281	23712	479
swath3.mps	640	1049	19946	23793	281	23712	433
tbfp-network.mps	42643	11572	244825	237023	*	237077	3225
thor50dday.mps	38456	*	*	*	*	*	*
timtab1.mps	328	250	744	812	502	751	175
tr12-30.mps	696	1321	3304	5668	4135	5688	696
traininstance2.mps	13397	13713	21531	21904	*	32415	15256
traininstance6.mps	10270	10282	16929	15868	11367	52302	10512
trento1.mps	57604	74004	40969	40642	*	40722	34730
triptim1.mps	*	*	*	*	*	*	*
uccase12.mps	*	*	*	*	*	*	*
uccase9.mps	116722	*	*	*	*	*	*
uct-subprob.mps	9495	7117	14877	16206	2416	16033	9294
unitcal_7.mps	150003	84117	*	*	*	*	31193
var-smallemery-m6j6.mps	59701	59539	28200	60575	*	61423	*
wachplan.mps	4655	5722	17255	17440	2080	16360	6012
bppc4-08.mps	267	307	5373	4064	3543	3194	203
brazil3.mps	224690	143249	228236	*	*	*	*
buildingenergy.mps	*	*	*	*	*	*	*
cbs-cta.mps	3915	4629	44014	19417	*	19132	3018
chromaticindex1024-7.mps	112861	*	*	*	*	*	*
chromaticindex512-7.mps	53453	*	*	*	*	*	24224
cmflsp50-24-8-8.mps	62624	29725	66646	66879	*	66483	46395
co-100.mps	1810	1432	27194	155300	*	156246	79949
cod105.mps	5043	10413	10806	*	2350	*	3517
comp07-2idx.mps	36630	19996	120086	140436	*	140131	124969
comp21-2idx.mps	17908	11221	77106	90612	3935	99479	83129
cost266-UUE.mps	1612	1782	3310	3582	4375	3626	1827
cryptanalysiskb128n5obj14.mps	83012	94390	*	125246	*	*	*
cryptanalysiskb128n5obj16.mps	83670	91007	*	142608	*	*	*
csched007.mps	1440	1902	7905	8245	1777	7794	603
csched008.mps	1012	1024	6211	6404	1265	6246	1647
cvs16r128-89.mps	28014	28833	26663	25872	*	28176	27912
dano3_3.mps	50506	*	*	*	*	*	*
dano3_5.mps	50506	*	*	*	*	*	*
decomp2.mps	2644	2574	7315	7555	3674	12070	5481
drayage-100-23.mps	4423	2050	15077	27251	2049	38281	6012
drayage-25-23.mps	4599	2189	14278	30007	1812	24166	6277
dws008-01.mps	313	246	1261	527	430	478	358
eil33-2.mps	233	241	1600	2129	151	2469	603
eilA101-2.mps	3325	3514	36453	79566	856	*	56899
enlight_hard.mps	29	29	29	29	29	29	29
ex10.mps	*	179784	*	*	*	*	*
ex9.mps	*	84528	*	*	*	*	*
exp-1-500-5-5.mps	602	1031	639	639	1311	639	457
fast0507.mps	27383	24272	196331	197020	*	197160	22921
fastxgemm-n2r6s0t2.mps	5691	3666	17063	22611	2964	19282	5782
fhnw-binpack4-4.mps	665	667	960	625	1230	615	994
fhnw-binpack4-48.mps	9521	7946	8830	4430	19723	4406	7236
fiball.mps	6688	8910	12339	79670	*	73548	4025
gen-ip002.mps	71	71	147	90	86	91	32
gen-ip054.mps	61	61	152	104	49	105	30
germanrr.mps	3879	4119	*	56995	*	*	9180
gfd-schedulen180f7d50m30k18.mps	*	*	*	*	*	*	*
glass-sc.mps	1589	2876	20767	21471	499	22452	610
glass4.mps	93	86	304	235	116	187	73
gmu-35-40.mps	1651	765	2876	1947	2183	2582	1494
gmu-35-50.mps	1955	1041	3785	3745	2992	4648	2405
graph20-20-1rand.mps	3843	2835	24081	*	2517	12187	4597
graphdraw-domain.mps	268	309	622	304	386	292	168
h80x6320d.mps	364	263	10938	8128	6955	8835	499
highschool1-aigio.mps	*	*	*	*	*	*	*
hypothyroid-k1.mps	10923	11179	26362	15838	7999	24741	4307
ic97_potential.mps	634	598	752	514	636	519	1101
icir97_tension.mps	3186	2145	3432	4216	3587	3547	3383
irish-electricity.mps	*	*	*	*	*	*	*
irp.mps	225	287	*	4075	139	23929	191
istanbul-no-cutoff.mps	11975	11678	82444	86299	*	80471	2781
k1mushroom.mps	81426	16692	18513	5144	*	5400	*
lectsched-5-obj.mps	154064	*	19026	65039	*	64952	45012
leo1.mps	1906	1042	10858	22634	10125	22835	275
leo2.mps	4766	2269	17501	36383	12522	36488	193
lotsize.mps	1918	1785	3498	3567	3148	3599	2010
mad.mps	37	87	97	154	52	226	40
map10.mps	91211	*	*	*	*	*	*
map16715-04.mps	*	*	*	*	*	*	*
markshare2.mps	39	39	97	110	62	107	39
markshare_4_0.mps	20	20	39	42	31	37	19
mas74.mps	129	129	516	566	307	563	23
mas76.mps	79	79	414	500	110	528	37
mc11.mps	1677	1894	3430	2643	4578	2592	1327
mcsched.mps	4708	5116	11932	11842	10099	11784	5820
mik-250-20-75-4.mps	75	75	75	75	75	75	75
milo-v12-6-r2-40-1.mps	3739	5212	26261	15404	4622	9373	2451
momentum1.mps	12355	8999	*	*	*	*	11537
mushroom-best.mps	*	57526	10307	46970	1123	*	*
mzzv11.mps	39840	13789	73458	88323	*	106101	3987
mzzv42z.mps	12448	9094	62218	56988	11379	80630	3614
n2seq36q.mps	4060	2892	77923	81065	3048	79541	4388
n3div36.mps	143	150	2276	5299	295	8577	37
n5-3.mps	1396	995	11634	11481	2012	11550	890
neos-631710.mps	31810	17901	*	*	*	*	*
neos-662469.mps	30106	23253	61914	63061	*	62946	50345
neos-787933.mps	2163	2246	*	9279	*	8700	2164
neos-827175.mps	171542	*	*	165678	*	165157	*
neos-848589.mps	2135	*	*	13758	*	*	2114
neos-860300.mps	1155	893	7698	7829	1321	7938	224
neos-873061.mps	110262	*	*	*	*	*	33
neos-911970.mps	129	133	3183	3363	214	3447	92
neos-933966.mps	158717	167025	169776	155596	*	158554	109968
neos-950242.mps	6928	7299	64874	80748	2590	5921	2630
neos-957323.mps	28302	12481	*	*	*	*	*
neos-960392.mps	9058	10795	203203	205651	*	206419	8805
neos17.mps	936	767	3430	1605	570	1379	700
neos5.mps	105	107	193	122	99	270	7
neos8.mps	712	429	4581	6209	553	9684	1124
neos859080.mps	4	4	93	91	83	90	4
neos-1122047.mps	8685	8825	*	*	*	*	9201
neos-1171448.mps	79780	71487	59813	6001	*	5843	18055
neos-1171737.mps	25462	21642	21740	2542	22777	2630	22927
neos-1354092.mps	90484	88911	67471	67230	*	61909	15903
neos-1445765.mps	6122	6555	28656	31982	10084	46889	5772
neos-1456979.mps	35773	8662	30198	35218	2142	35123	3378
neos-1582420.mps	3670	2964	47584	63690	6191	64109	4293
neos-2075418-temuka.mps	*	*	*	*	*	*	*
neos-2657525-crna.mps	360	200	627	699	305	678	292
neos-2746589-doon.mps	120878	36276	*	271882	*	*	14163
neos-2978193-inde.mps	928	2709	37226	64529	2335	64472	833
neos-2987310-joes.mps	14222	13281	23023	25572	*	26282	15458
neos-3004026-krka.mps	6660	6660	6305	6305	*	6305	11610
neos-3024952-loue.mps	3645	3863	13076	4545	8948	5767	3073
neos-3046615-murg.mps	169	169	235	235	455	235	169
neos-3083819-nubu.mps	2678	2409	8224	11544	2240	15352	1681
neos-3216931-puriri.mps	*	34396	45197	45797	9782	50236	*
neos-3381206-awhea.mps	1584	1774	2196	2312	1978	2227	2002
neos-3402294-bobin.mps	1066	1023	3374	*	461	*	1134
neos-3402454-bohle.mps	5481	3457	*	*	*	*	*
neos-3555904-turama.mps	6076	1607	*	*	*	*	4329
neos-3627168-kasai.mps	1633	1900	4127	3905	5382	5776	1217
neos-3656078-kumeu.mps	20525	17539	113007	110161	*	109498	9260
neos-3754480-nidda.mps	291	430	2202	974	635	1035	231
neos-3988577-wolgan.mps	40469	24658	*	*	*	*	*
neos-4300652-rahue.mps	85331	17034	48186	*	*	*	26589
neos-4338804-snowy.mps	683	692	441	441	792	441	1681
neos-4387871-tavua.mps	2292	2471	9202	6459	3000	26601	3427
neos-4413714-turia.mps	27839	10821	*	*	*	*	16425
neos-4532248-waihi.mps	3191	982	*	*	*	*	7981
neos-4647030-tutaki.mps	11605	12066	18514	18452	*	18167	*
neos-4722843-widden.mps	14983	10825	*	30597	*	37043	*
neos-4738912-atrato.mps	4027	5190	26204	26796	27602	26215	5533
neos-4763324-toguru.mps	302123	71563	*	*	*	*	*
neos-4954672-berkel.mps	962	965	3574	3792	3055	4385	670
neos-5049753-cuanza.mps	*	*	*	*	*	*	*
neos-5052403-cygnet.mps	*	*	*	*	*	*	*
neos-5093327-huahum.mps	19617	20575	*	*	*	*	*
neos-5104907-jarama.mps	*	*	*	*	*	*	*
neos-5107597-kakapo.mps	6191	6969	14446	11175	*	10175	8147
neos-5114902-kasavu.mps	*	*	*	*	*	*	*
neos-5188808-nattai.mps	26125	12571	24862	46541	*	*	9429
neos-5195221-niemur.mps	43996	14654	21534	*	*	*	13985
net12.mps	3929	5043	29848	92171	4716	92870	1160
netdiversion.mps	*	*	*	*	*	*	*
nexp-150-20-8-5.mps	661	650	2707	5219	11536	6470	845
ns1116954.mps	5600	12858	441155	24617	*	81870	19024
ns1208400.mps	3263	2398	25447	25859	2281	26724	4545
ns1644855.mps	*	*	*	*	*	*	*
ns1760995.mps	*	31060	*	*	*	*	*
ns1830653.mps	18120	16096	18666	19139	4744	19311	11198
ns1952667.mps	68	68	11145	18491	87	24957	68
nu25-pr12.mps	1850	1642	6699	26414	3099	26281	6165
nursesched-medium-hint03.mps	156192	36390	160684	169581	*	168361	35869
nursesched-sprint02.mps	16279	8259	47487	46400	3100	46185	46907
nw04.mps	438	313	12328	56821	130	*	94
opm2-z10-s4.mps	*	*	28984	*	*	*	*
p200x1188c.mps	578	579	1603	1061	1177	1018	1244
peg-solitaire-a3.mps	11502	10055	31045	30794	6902	30915	19201
pg.mps	1566	1567	3628	3730	8714	4477	1552
pg5_34.mps	1684	1999	8040	8664	3631	8641	3022
physiciansched3-3.mps	*	*	*	*	*	*	*
physiciansched6-2.mps	*	*	*	*	*	*	*
piperout-08.mps	10035	8071	77316	78677	13883	79658	10936
piperout-27.mps	13212	10491	93333	97329	*	98047	19730
pk1.mps	56	56	234	256	229	220	56
proteindesign121hz512p9.mps	963	905	13935	19537	*	*	1311
proteindesign122trx11p8.mps	606	652	11270	10568	2918	10460	713
qap10.mps	43368	*	47037	42295	*	51073	8507
radiationm18-12-05.mps	40126	50952	*	*	*	*	*
radiationm40-10-02.mps	163087	*	*	*	*	*	*
rail01.mps	*	*	*	*	*	*	*
rail02.mps	*	*	*	*	*	*	*
rail507.mps	24907	20067	195912	196516	*	197922	23777
ran14x18-disj-8.mps	1840	1657	3587	3867	3889	3821	2611
rd-rplusc-21.mps	475	412	342	913	640	840	517
reblock115.mps	2456	5185	11934	2566	4122	2627	3220
rmatr100-p10.mps	3409	4706	35091	15813	1672	17698	501
rmatr200-p5.mps	65929	85034	*	*	*	*	309
rocI-4-11.mps	9498	13144	46230	54614	7263	42979	20292
rocII-5-11.mps	6064	1315	3874	2342	1884	2382	1615
rococoB10-011000.mps	20776	1353	21938	21425	3977	21581	11982
rococoC10-001000.mps	3941	1511	7374	13936	5032	14324	522
roi2alpha3n4.mps	24697	10346	6264	32500	39804	10864	9391
roi5alpha10n8.mps	12459	6064	13999	*	*	132274	*
roll3000.mps	1815	2155	12717	13269	1892	13997	3166
s100.mps	*	*	*	*	*	*	*
s250r10.mps	*	*	*	*	*	*	*
satellites2-40.mps	73896	51346	19366	213784	*	21851	2498
satellites2-60-fs.mps	47644	41964	18033	181057	*	16223	2921
savsched1.mps	*	*	*	*	*	*	*
sct2.mps	6949	2983	27287	26895	7397	27005	3571
seymour.mps	7345	9261	22067	24986	2502	24940	15189
seymour1.mps	7345	9261	22067	24986	2502	24940	15189
sing44.mps	261483	*	*	*	*	*	*
sing326.mps	220981	*	*	*	*	*	*
snp-02-004-104.mps	*	*	*	*	*	*	*
sorrell3.mps	1397	1356	1772	1369	1335	1368	1736
sp150x300d.mps	318	310	577	479	484	475	307
sp97ar.mps	3950	3573	40951	50077	2571	49995	14935
sp98ar.mps	20342	10799	39116	52886	2519	52395	1967
splice1k1.mps	35351	17482	34404	48443	*	32872	16224
square41.mps	*	*	*	*	*	*	*
square47.mps	*	*	*	*	*	*	*
supportcase10.mps	*	*	*	*	*	*	*
supportcase12.mps	*	*	*	*	*	*	*
supportcase18.mps	1787	1077	22628	27455	652	37010	499
supportcase19.mps	*	*	*	*	*	*	*
supportcase22.mps	*	2431	*	*	*	*	*
supportcase26.mps	294	313	365	409	450	409	540
supportcase33.mps	4216	3176	44224	140227	*	87128	5053
supportcase40.mps	100160	38705	*	175363	*	181878	21721
supportcase42.mps	*	*	*	*	*	*	*
supportcase6.mps	9576	3895	*	*	*	*	931
supportcase7.mps	154110	24378	*	*	*	*	*
swath1.mps	640	1049	19946	23793	281	23712	479
swath3.mps	640	1049	19946	23793	281	23712	433
tbfp-network.mps	42643	11572	244825	237023	*	237077	3225
thor50dday.mps	38456	*	*	*	*	*	*
timtab1.mps	328	250	744	812	502	751	175
tr12-30.mps	696	1321	3304	5668	4135	5688	696
traininstance2.mps	13397	13713	21531	21904	*	32415	15256
traininstance6.mps	10270	10282	16929	15868	11367	52302	10512
trento1.mps	57604	74004	40969	40642	*	40722	34730
triptim1.mps	*	*	*	*	*	*	*
uccase12.mps	*	*	*	*	*	*	*
uccase9.mps	116722	*	*	*	*	*	*
uct-subprob.mps	9495	7117	14877	16206	2416	16033	9294
unitcal_7.mps	150003	84117	*	*	*	*	31193
var-smallemery-m6j6.mps	59701	59539	28200	60575	*	61423	*
wachplan.mps	4655	5722	17255	17440	2080	16360	6012

## Footnotes

1 We would like to point out that to prove the theorem, it suffices to choose *any* non-basic index with negative reduced cost in the support of $y^0$ as the entering index. The fact that *f* has the most negative reduced cost among such indices will only be used for the results in the next section.
